# Association screening for genes with multiple potentially rare variants: an inverse-probability weighted clustering approach

**DOI:** 10.1186/1753-6561-5-S9-S106

**Published:** 2011-11-29

**Authors:** Ying Liu, Chien Hsun Huang, Inchi Hu, Shaw-Hwa Lo, Tian Zheng

**Affiliations:** 1 Department of Statistics, Columbia University, New York, NY 10027, USA; 2 Department of Information Systems, Business Statistics, and Operations Management (ISOM), Hong Kong University of Science and Technology, Kowloon, Hong Kong

## Abstract

Both common variants and rare variants are involved in the etiology of most complex diseases in humans. Developments in sequencing technology have led to the identification of a high density of rare variant single-nucleotide polymorphisms (SNPs) on the genome, each of which affects only at most 1% of the population. Genotypes derived from these SNPs allow one to study the involvement of rare variants in common human disorders. Here, we propose an association screening approach that treats genes as units of analysis. SNPs within a gene are used to create partitions of individuals, and inverse-probability weighting is used to overweight genotypic differences observed on rare variants. Association between a phenotype trait and the constructed partition is then evaluated. We consider three association tests (one-way ANOVA, chi-square test, and the partition retention method) and compare these strategies using the simulated data from the Genetic Analysis Workshop 17. Several genes that contain causal SNPs were identified by the proposed method as top genes.

## Background

Rare variants are common on the genome and have long been speculated to be involved in the etiology of most human disorders [1]. In the 2000s, a large number of genome-wide association studies (GWAS) were conducted using relatively more common single-nucleotide polymorphisms (SNPs) (with minor allele frequency [MAF] > 5%). Most of the common variants identified in these studies have borderline odds ratios and can explain only a small fraction of susceptibility to a disease [2]. As a result, there has been increasing interest in the study of rare variants for complex diseases. This concern has also been fueled by advancements in sequencing technology. In particular, the availability of such technology has directly led to the implementation of the 1000 Genomes Project (http://www.1000genomes.org/), in which 1,000 genomes from individuals of different ethnic backgrounds were sequenced, consequently leading to the identification of a large number of rare variants (SNPs) with MAF < 1% and some very rare variants with MAF < 0.5%. Because of these low MAFs, association methods developed for common variants have limited efficiency for mapping rare variants in population studies. For these methods to have adequate power to detect individual rare variants, the sample size needs to increase substantially as the MAF decreases.

It is also more likely for a rare variant to contribute to the susceptibility of a disease as part of a group of rare variants in the same gene or pathway. Therefore grouping or collapsing rare variants is the most feasible option to improve efficiency in studying rare variants. Usually, the grouping is constructed on the basis of functional relevancy, physical proximity, or both. Once rare variants have been grouped, their genotypic information is combined, or collapsed, into a usually univariate score, and the association between the group of rare variants and the disease is then studied using the association between the univariate score and the disease traits. See Asimit and Zeggini [2] and Dering et al. [3] for excellent reviews of different methods for rare variant association analysis, including single-marker, multimarker, and various collapsing strategies. A popular alternative to collapsing genotypic information is to combine single-SNP statistics.

In this paper, we consider a gene-based association analysis for rare variants. This is equivalent to grouping based on the gene affiliation of SNPs. We propose using a clustering-based method for collapsing genotypic information of multiple SNPs within each gene. The clustering is based on an inverse-probability weighted sum of genotypic differences that highlights the variation at rare variant loci. Association between the collapsed partition label and the disease traits can then be readily evaluated using single-marker association methods, such as one-way analysis of variance (ANOVA), a chi-square test, and the partition retention method [4,5]. We apply our approach to the simulated data of the Genetic Analysis Workshop 17 (GAW17) without knowledge of the simulation models. After the workshop, a comparison of our results with the simulation answers led to interesting observations regarding both the method and the simulated data. We discuss these observations in the Results section.

## Methods

### Data set

The simulated data set of GAW17 is a combination of real sequence data and simulated phenotypes. An exome of 3,205 autosomal genes, corresponding to 24,487 SNPs, was selected. Sequences of these SNPs were obtained from the 1000 Genomes Project on 697 unrelated subjects. SNPs with missing values were imputed using fastPhase. A majority of the SNPs (74%) were rare variants (MAF < 1%). Two hundred phenotype sets were simulated based on these common genotype data. Each simulated unrelated-individual data set has three quantitative trait values (Q1, Q2, Q4) and the Affected status *Y*, with 209 case subjects and 488 control subjects. Gene information and SNP information were provided. Especially, whenever available, SNPs were labeled as synonymous or nonsynonymous [6].

### Gene-based grouping and collapsing of SNP genotypes

We propose to evaluate an individual gene’s association with disease traits. SNPs within a gene are grouped for the association analysis. Our main focus is a collapsing strategy for multiple-SNP genotypes within a gene. We propose to create partitions of individuals (or observed genotypes) based on their genotypic differences evaluated by inverse-probability weighted similarity scores. It is easier to start with considering alleles at a single SNP locus first. For two individuals, we can count when they have the same alleles or different alleles. When the MAF is small, the chance of having a random match for the major allele is high. On the other hand, if a rare variant is involved in the etiology of a disease, then the case subjects are more likely to have the same rare variants than the control subjects are. Therefore for rare variant association analysis we want to overweight the allelic or genotypic similarity for the minor alleles but not that for the major alleles.

We use the inverse-probability weighted similarity score, as defined in Table 1. This score has a mean similarity 0, which is also a desirable property. The allelic similarity can be straightforwardly generalized to the genotypic similarity scores in Table 2. For example, an individual 1 with genotype *aa* and an individual 2 with genotype *Aa* will have one match (*a*, *a*) and one mismatch (*a*, *A*). Because *a* is the minor allele, the (*a*, *a*) match will dominate the (*a*, *A*) mismatch, and these two individuals will have a high similarity score. Such a weighting scheme implicitly assumes that individuals with the same rare variants will be clustered together for association analysis with the disease outcomes.

**Table 1:** Inverse probability similarity measure: allelic similarity scores

Individual 2	Individual 1	
	
	*a*	*A*
*a*		
*A*		

*p_a_* is the population frequency of minor allele *a*.

**Table 2:** Inverse probability similarity measure: genotypic similarity scores

Individual 2	Individual 1		
	
	*aa*	*aA*	*AA*
*aa*			
*aA*			
*AA*			

*p_a_* is the population frequency of minor allele *a*.

We denote the genotypic similarity score between two individuals *i* and *j* at SNP *k* by sim(*i*, *j*; *k*). For a given gene *G*, the similarity between *i* and *j* is defined as the sum of the similarity scores on SNPs within the gene:(1)

**Figure 1:**
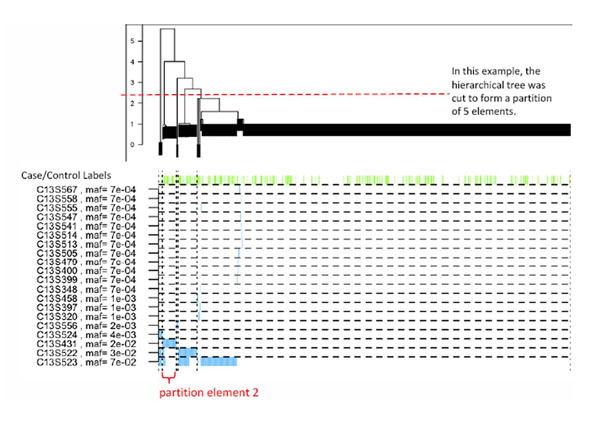
**Clustering of individuals using nonsynonymous SNPs for *FLT1.*** Each row is a SNP, and each column is an individual. Green vertical bars indicate case subjects. Genotype *aA* is plotted in blue, and genotype *AA* is plotted in white (*a* is the minor allele); the genotype *aa* was not observed. The partitions of the 697 individuals are indicated by dotted lines. Partition element 2 is driven by similarity on SNP C13S431 but not on the more common SNPs C13S522 and C13S523.

For the 697 individuals, pairwise similarity scores, the sim(*i*, *j*), are evaluated first and are then converted to a distance measure using the transformation:(2)

where *a* is a normalizing constant such that the distance calculated at each gene is bounded by *e*^20^. We then apply hierarchical clustering using Ward’s method [7] and partition individuals into groups by cutting the hierarchical clustering tree into a prespecified number of groups (we consider partition sizes of 5 to 10). See Figure 1 for an example using *FLT1*. We also take advantage of the synonymy information about the SNPs by carrying out two separate analyses using nonsynonymous SNPs only or every SNP in a gene.

### Partition-based association analysis

After obtaining the partition of individuals, for each gene we tested the association between the partition indexes obtained from the SNPs in that particular gene and the disease phenotypes. For the disease status *Y*, we considered one-way ANOVA, the chi-square test of independence, and the partition retention method [4]. For continuous-valued disease outcomes Q1, Q2, and Q4, we considered one-way ANOVA and the partition retention method.

The partition retention method is based on association measure *I* defined between an outcome variable *Y* and a partition Π. More specifically,(3)

where *n_i_* is the number of individuals in partition element *i* and  is the sample mean of element *i*.  and *s* are the sample mean and the standard deviation of all *n* individuals, respectively. Under the null hypothesis, *I* asymptotically converges to a weighted sum of chi-square distributions with 1 degree of freedom and therefore has mean 1. The partition retention method is more robust to sparse partition than the chi-square test and can be applied to both dichotomous disease status and continuous-valued traits [4]. Intuitively, the *I* in the partition retention method evaluates the amount of influence a particular gene has on the disease phenotypes.

*p*-values for the ANOVA test and the chi-square test are derived from corresponding asymptotic distributions. To address the multiple testing issue, we control the family-wise error rate using the conservative Bonferroni correction. For the evaluation using the partition retention *I*, we simply chose the top 0.1% of genes for each trait. A further examination of results from chromosome 4 revealed that, by using a cutoff of the top 0.1%, only 15 of the 200 replicates returned any null gene (a family-wise type I error rate), which suggests that the top 0.1% is a reasonable threshold. In practice, we suggest evaluating *p*-values using permutations and controlling the false discovery rate in order to have better sensitivity to real genetic signals.

## Results

Because we have 200 simulation sets, for each gene we counted the number of times it was selected (either in the top 0.1% for *I* using the partition retention method or significant by Bonferroni correction for ANOVA and the chi-square test) for each trait for each method. We also compared the effects of partition sizes (results not shown). The significance varied between different partition sizes, and the partition size that corresponded to the most significant results also changed from simulation to simulation. Therefore we used the average count across six partition sizes (from 5 to 10) to rank genes. By visually examining the average counts (not shown), we observed that Q1 had strong genetic signals and that Q2 and Affected status were harder to map. For Q4, the one-way ANOVA identified many noncausal genes, or false positives, to which the partition retention method was relatively more immune.

**Figure 2:**
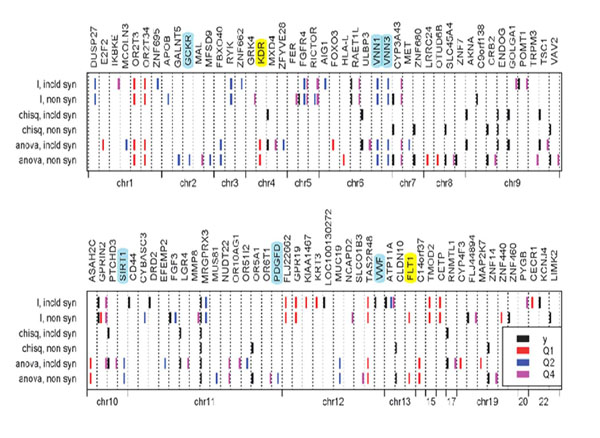
**Top ten genes identified by each of the methods and for each of *Y*, Q1, Q2, and Q4**. Ninety-one genes are shown, displayed by chromosome. Genes with causal SNPs are highlighted (yellow for Q1 and blue for Q2).

Figure 2 summarizes the results from the 200 simulations. The top 10 genes for each method and each trait are plotted by chromosome. Note that for Q2 the top 10 genes are identified less than 25% of the time and that the six genes that contain “answers” or causal genes are identified as top genes but with less than 5% probability, with the exception of *VNN1*, which is identified by the partition retention method 22% of the time. Two genes for Q1 (*FLT1* and *KDR*) are identified in more than 50% of the simulated replicates. It is interesting to note that excluding synonymous SNPs led to better identification of *FLT1* and had less effect on identification of *KDR*.

To better understand the “consistent false positives” problem that arose during GAW17, we studied several consistent false-positive genes identified by our methods. All of them were found to be significantly associated with multiple causal SNPs. See Table 3 for an example between the gene *OR2T3* on chromosome 1 and a causal SNP at C13S523.

**Table 3:** Association between a consistent false-positive gene (*OR2T3*) and a causal SNP at C13S523

C13S523 genotype (*p* = 1.8 × 10^−18^ by Fisher’s exact test)	Partition based on SNPs of *OR2T3*				
	
	1	2	3	4	5
1	41	29	3	9	11
2	525	59	5	8	7

We further investigated the relation between power to detect (probability of true positive) and the effect size of a gene. The effect size for each SNP is provided by Almasy et al. [6]. For each gene, we define its total effect size as:(4)

where *β_i_* is the effect size *β* used in the simulation model for SNP *i*, which is 0 for noncausal SNPs.

**Figure 3:**
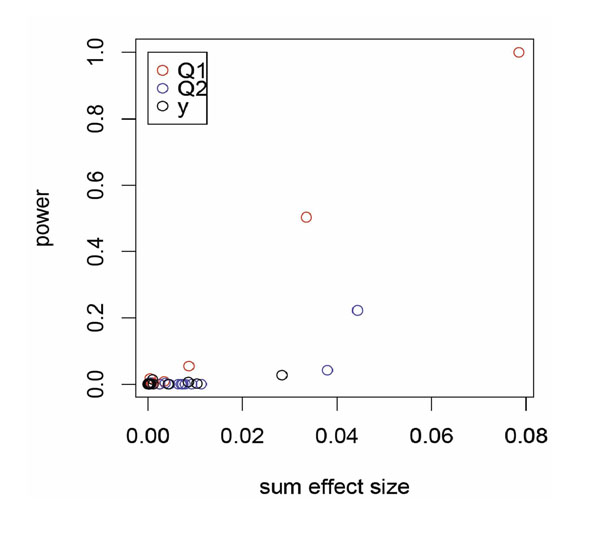
**Power to identify a causal gene versus effect size**. For each trait, we plot the power to detect using the best performing method against the effect size used in the simulation model. That is, we plot the one-way ANOVA with Bonferroni correction for Q1 and *Y*, and the *I* from the partition retention method for Q2. The gene-wise effect size is defined as the sum of SNP-wise MAF × causal SNP effect in the simulation model.

Figure 3 plots the frequencies of each gene with causal genes identified by the best performing method for each trait against the gene-wise effect size, that is, the one-way ANOVA with Bonferroni correction for Q1 and *Y* and the *I* from the partition retention method for Q2. The power of our approach suffers greatly for extreme rare variants if the effect size does not scale up as MAF drops.

## Discussion and conclusions

In this paper, we propose a novel strategy for gene-based association analysis for genes with multiple potentially rare variants. The inverse-probability weighted clustering approach automatically adjusts weights for rare variants and overweights their genotypic variation when comparing individuals for an association study. Individuals are first partitioned on the basis of their genetic similarity on multiple SNPs in a gene, and this partition is then used to calculate association between a gene and a disease trait.

We also considered several association scores and the effect of including synonymous variants. Different methods seem to focus on nonoverlapping signals, which suggests a multimethod approach for future association studies. From our results, we can conclude that our method gains power by considering multiple rare variants in a gene, as illustrated in Figure 1 for one of our identified causal genes. It is probably beneficial to consider synonymous and nonsynonymous SNPs in future practice. Filtering out synonymous SNPs corresponds to a weight of 0 being assigned to synonymous SNPs and a weight of 1 being assigned to nonsynonymous SNPs, which can be extended to a smoother weighting scheme as a possible future direction.

For this simulation study, we used asymptotic *p*-values and the conservative Bonferonni correction because we needed to analyze 200 sets of data. In practice, we suggest evaluating *p*-values using permutations and controlling the false discovery rate in order to have better sensitivity to real genetic signals. Population information is provided with the simulated data. Some consistent false positives may have resulted from confounding due to population admixture. We recommend using existing methods, such as Eigensoft [8], to adjust for population stratification in real applications when applying our method. It should be pointed out that algorithms such as Eigensoft [8] may convert the original discrete genotype data to continuous values, which requires modification to the similarity measure defined in Table 1.

## Competing interests

The authors declare that they have no competing interests.

## Authors’ contributions

TZ conceived the study and coordinated the project activities. TZ and YL designed the research and performed the statistical analysis. CHH preprocessed the data. TZ, YL, CHH, SHL and IH discussed and interpreted the results and participated in the preparation of the manuscript. All authors read and approved the final manuscript.

## References

[B1] Pritchard JK Are rare variants responsible for susceptibility to complex diseases? Am J Hum Genet 2001 69 124 137 10.1086/321272 11404818 PMC1226027

[B2] Asimit J Zeggini E Rare variant association analysis methods for complex traits Annu Rev Genet 2010 44 293 308 10.1146/annurev-genet-102209-163421 21047260

[B3] Dering C Pugh E Ziegler A Statistical analysis of rare sequence variants: an overview of collapsing methods Genet Epidemiol 2011 3 Suppl 8 12 17 10.1002/gepi.20643 PMC3277891 22128052

[B4] Chernoff H Lo SH Zheng T Discovering influential variables: a method of partitions Ann Appl Stat 2009 3 1335 1369

[B5] Zheng T Chernoff H Hu I Ionita-Laza I Lo SH HHS Lu, B Scholkopf, H Zhao Discovering influential variables: a general computer intensive method for common genetic disorders Handbook of Computational Statistics: Statistical Bioinformatics 2010 New York, Springer

[B6] Almasy LA Dyer TD Peralta JM Kent JW Jr. Charlesworth JC Curran JE Blangero J Genetic Analysis Workshop 17 mini-exome simulation BMC Proc 2011 5 suppl 9 S2 22373155 10.1186/1753-6561-5-S9-S2 PMC3287854

[B7] Dasgupta A Sun YV Konig IR Bailey-Wilson JE Malley J Brief review of regression-based and machine learning methods in genetic epidemiology: the GAW17 experience Genet Epidemiol 2011 X suppl X X X 10.1002/gepi.20642 PMC3345521 22128059

[B8] Price AL Patterson NJ Plenge RM Weinblatt ME Shadick NA Reich D Principal components analysis corrects for stratification in genome-wide association studies Nat Genet 2006 38 904 909 10.1038/ng1847 16862161

